# The health and wellbeing of young people in sub-Saharan Africa: an under-researched area?

**DOI:** 10.1186/1472-698X-13-11

**Published:** 2013-02-13

**Authors:** Caroline W Kabiru, Chimaraoke O Izugbara, Donatien Beguy

**Affiliations:** 1 African Population and Health Research Center, Inc., APHRC Campus, Manga Close, off Kirawa Road, P. O. Box 10787–00100, Nairobi, Kenya

**Keywords:** Youth, Sub-Saharan Africa, Research

## Abstract

A third of sub-Saharan Africa’s (SSA) population comprises persons aged 10–24 years. These youth are growing up in a context marked by pervasive poverty, limited educational opportunities, high HIV/AIDS prevalence, widespread conflict, and weak social controls. Published research on the broad issues that affect youth health and wellbeing in SSA is limited and centers heavily on sexual and reproductive health. In this commentary, we provide a broad overview of sub-Saharan African youth, highlight research gaps with respect to youth health and wellbeing, and describe potential avenues to develop the region’s research capacity on youth health and wellbeing.

## Background

Persons aged 10–24 years comprise about 33% of the population in sub-Saharan Africa [1]. While other countries in the developing world will experience a decline in the proportion of their population that comprises youth, sub-Saharan Africa (SSA) will continue to experience an increase [2]. The youth bulge in many African countries stems from high fertility and improvements in child health. As these youths become older and have fewer children, an increase in the productive labor force (15–64 years) relative to dependent populations of children under age 15 and older adults can provide an opportunity for rapid economic growth, otherwise known as the “demographic dividend.” [3] Evidence shows that fertility rates in the region have declined in the past three decades, albeit more slowly in recent years [4]. However, for the region to take advantage of its youthful population and reap the demographic dividend stemming from a large working age population, African governments must ensure that youth have adequate opportunities and skills to engage meaningfully in the social and economic sectors, and adopt healthy behaviors [3].

Majority of sub-Saharan African youth are growing up in a context of widespread poverty, high rates of unemployment, rapid urbanization, often limited educational opportunities, and rapid socio-cultural transformations characterized by weakening social controls and breakdown of traditional norms [5-8]. These challenges have far-reaching implications for the health and wellbeing of youth and for the potential contributions of youthful populations to the economies of African nations.

Outside of sexual and reproductive health issues affecting young people, limited research has been conducted on the broader context of youth health in SSA. Strengthened capacity to generate rigorous scientific evidence to inform policies and programs designed to improve the health and wellbeing of young people is therefore needed. In this commentary, we highlight some of the major influences on young people’s (10–24 years) health and wellbeing in SSA; explore some of the gaps in research on young people; and propose avenues to develop the region’s research capacity on young people’s health and development. The research gaps are intended to be illustrative of potential areas of research on the broader context of youth health and wellbeing.

### Influences on youth health and development in Africa and implications for research

#### Education and schooling

Education has been positively linked to good health, economic growth, and fewer social conflicts [9,10]. In Africa, however, millions of youth remain without access to formal education. While school enrolment has expanded dramatically in most SSA countries, literacy rates among 15–24 year olds (72.4%) remain the lowest globally [11]. This situation has important implications for the continent. A large number of under-educated youth in a context of rapid population growth and poor economies [7] is ominous for development, health and security in the region. Many sub-Saharan African youth join the labor force without adequate educational and professional preparation. Child labor in the region is among the highest in the world – evidence from 29 African countries shows that, on average, 35% of children under the age of 15 work [2]. Early entry into employment has been associated with poor labor market experiences and reduced earning potential [12]. Overall, uneducated and unemployed youth are easily manipulated and exploited, and form easy recruitment targets for criminal gangs, traffickers, and armed insurgent movements [13]. While the long-term impacts of limited schooling are well-researched [14], we know little about the most effective ways to keep youth in school. Evidence is needed on innovative approaches to increase access to basic education, address child labor, and to improve the quality of teaching particularly in resource-poor settings.

#### Urbanization

Although sub-Saharan Africa is currently the least urbanized region in the world, the region is experiencing the world’s highest urban growth rate [15]. Over one-third of sub-Saharan Africans currently reside in urban areas. However, many African governments are unable to adequately cope with the swelling number of urban residents resulting in rapid ‘slumization’. Estimates indicate that well over two-thirds of urban population residents in sub-Saharan live in slum settlements characterized by ubiquitous poverty, lack of basic infrastructure, a dearth of socio-economic opportunities, excessive overcrowding, extreme deprivation, and enduring marginalization [16]. Sommers [17] notes that urban youth are at the heart of the current demographic transformations occurring in Africa – youth in search of employment, education and other opportunities constitute the bulk of migrants to African cities [7,18]. While urbanization can—and does—stimulate economic growth [19], radical improvements in local governance and infrastructure are urgently needed to maximize the promises of urban growth in Africa, and effectively support youth to benefit from the process.

Existing evidence underscores some key consequences of urbanization. For example, Twagirumukiza and colleagues [20] found a higher prevalence of hypertension among urban residents compared to rural dwellers in several African countries, which they attributed to lifestyle differences. Likewise, Adamo and colleagues in a study comparing Kenyan and Canadian youth found a higher prevalence of overweight and obesity among urban youth compared to their peers in rural settings [21]. With respect to mental health, Swahn and colleagues [22] found that 20% of youth in a study conducted among a convenience sample of 14–24 year olds living in the streets and slums of Kampala, Uganda had attempted suicide. The unique vulnerability of urban youth suggests that evidence is needed on the epidemiology of young people’s health in urban areas and potential avenues to address their health needs.

#### Globalization

Globalization, a dominant feature of this century [23], has promoted the transnational movement of ideas, cultures, knowledge, and technology and ensured greater access to information, education, and opportunities [24]. However, globalization has also contributed to massive economic restructuring and structural adjustments leading to the shrinking capacity of many governments to support young people [25]. Globalization has also increased the role of peers and western media in the socialization of African youth and exposed them to sexually-explicit materials, illicit drugs, and cultures and lifestyles, which they are ill-prepared to deal with [6,26,27]. The exclusion of African youth from power, work, and education, among others may force them to explore alternatives spaces of socialization including the Internet [28]. Although access to the Internet increases young people’s access to information and provides a useful platform for social interaction, young people’s active use of the Internet and social media also increases their vulnerability to cyberbullying, pornography, and sedentary lifestyles, among other risks [29]. Research is needed on the impacts of globalization on the health and development of African youth. For example, research on the potential benefits and impacts of the Internet would be beneficial given young African’s growing exposure to the Internet.

#### The HIV/AIDS pandemic

HIV/AIDS is among the top public health concerns and causes of mortality and morbidity in Africa, with 70% of all AIDS deaths currently occurring in the region [30]. In 2011, there were an estimated 1.8 million new HIV infections in the region: the world’s highest [30]. Generally, youth in Africa are at the epicenter of the HIV/AIDS epidemic – in 2010 15–24 year olds accounted for 42% of new HIV infections with young women particularly at risk [31,32].

The marked emotional, sexual, and psychological transformations associated with adolescence increase experimentation with behaviors and practices that can have longstanding implications for their health and wellbeing [32,33]. Unfortunately, many young people in Africa still lack comprehensive and correct HIV knowledge [34] and their access to sexual and reproductive health services is often limited [23]. Although young people’s sexual and reproductive health has received significant attention from researchers, further evidence is needed on ways to address the sexual and reproductive health challenges that young people face. For example, existing research shows low levels of HIV testing among youth, particularly among males [35-37]. Given the high vulnerability of young people to HIV [31], research on cost-effective strategies to improve young people’s access to HIV testing and counseling is needed. There is also need to generate more contextual evidence on the social, cultural, economic and structural factors that increase young people’s vulnerability, particularly young females, to HIV and other sexually transmitted infections and approaches to address these factors.

#### War and conflict

Many SSA countries have been embroiled in war and conflict in the post-colonial period [38]. More than half of the region’s countries are experiencing or have experienced conflict, with the Horn of Africa, the Great Lakes region, the former Portuguese colonies of Angola and Mozambique, and the Upper Guinea/Mano River territory in West Africa [39] being the four primary zones of conflict. A blend of factors — including high unemployment especially of youth, international economic policies, power politics, frustration from decades of living under authoritarian and corrupt regimes — have fueled conflicts. Other contributory factors include the authoritarian legacies of colonialism, lack of peaceful means for regime or leadership change, and the politicization of ethnicity [40]. African wars and conflicts have been devastating in their effects – over 9 million refugees and internally displaced people have been reported in the last one decade. Youth form a substantial proportion of those killed, raped, or injured in situations of conflicts [23].

The literature on youth bulges in Africa also identifies a security threat associated with a large number of underemployed youths [40]. Combatants in many armed conflicts in the region are youth [13]. Their feelings of exclusion, desires to be valued, and sense of invulnerability make it easy to mobilize them into armed conflicts [13]. Abdullah [41], for example in his examination of conflict in Sierra Leone argues that the youth involved in conflict are largely ‘unemployed and unemployable, live by their wits [and] have one foot in what is generally referred to as the informal or the underground economy. They are prone to criminal behavior, petty theft, drugs, drunkenness, and gross indiscipline’.

Although armed conflicts in SSA continue in several regions, there is limited research on the impacts of conflict on young people [39]; the involvement of girls and female youths in conflict – not just as victims, but as combatants and active participants in wars [42]; and reintegration of youth affected by conflicts into regular civilian life [39,42]. Also lacking is evidence on the unique social and economic needs of youth in post-conflict situations.

#### The lack of specialized health care to address youths’ needs

Youth in Africa face substantial barriers in accessing health services: age- and marital status-related barriers; lack of confidentiality; fear of mistreatment; inconvenient hours and locations of facilities; high costs of services; and limited knowledge of available services [43]. With respect to marital status, the use of sexual and reproductive health services is often a challenge for unmarried young people in contexts where pre-marital sexual activity is proscribed [43,44]. Youth-friendly health facilities have potential to ensure that services reach young people and that their unique health needs are met [43]. However, according to the World Health Organization (WHO), most SSA countries have a dearth of youth-friendly health services and inadequate policies to address adolescent health needs [45]. For example, in South Africa, one of the better-endowed countries in the region, young people aged 13 and above are typically attended to in the adult health care system by health professionals who lack specialized training to handle the needs of young people [46]. Research is needed on the attributes and the availability of youth-friendly services. Further, although several studies have examined reproductive health service utilization among youth [44,47] additional studies that examine health-seeking behaviors for other health needs among young people and that investigate various strategies to increase young people’s access to health services will also provide a strong evidence base for policy and programmatic efforts to improve the health of young people.

## Discussion

Sub-Saharan African youth are growing up in very challenging contexts. Yet, the ramifications of major influences on youth development in SSA have yet to be fully understood. Research evidence is needed to fully understand the issues that confront young people in contemporary SSA and develop programs and policies to mitigate the difficult circumstances facing them. However, the literature on youth in the region is quite limited and focuses largely on sexual and reproductive health, primarily in light of high fertility rates and the HIV pandemic. Yet, recent studies demonstrate that young people in SSA are at risk for other factors that impact negatively on their health and wellbeing including, poor dietary habits and physical inactivity [48], poor mental health [22,49], and injuries [50,51], among others. For example, Jenkins and colleagues [49] observed a 5.8% prevalence of common mental disorders among 16–29 year olds in a population-based study in Kenya.

Although there is substantial literature on multiple drivers of adolescent health and wellbeing in the global north [52], the unique context in which many African youth are experiencing the transition of adulthood means that this literature may only provide a partial picture of what is needed to inform policies and programs geared towards African youth. High-quality research capacity within the continent is vital if SSA is to develop effective evidence-based policies and programs to ensure the wellbeing of her youth. Unfortunately, SSA’s capacity to produce scientific knowledge is greatly impeded by multiple factors including reduced scientific output in many of her universities due to heavy teaching loads [53], significant emphasis on consultancies to supplement incomes [54], inability of governments to fund research, and loss of academics and researchers to institutions in the global north or non-research sectors [55,56]. The consequences of this situation are quite telling – a recent review of the region’s contribution to scientific knowledge as measured by papers published in various international databases shows that the region contributes to less than 1% of the world science production [57]. The low research outputs therefore undermine the capacity of African countries to draw on locally-owned and relevant research evidence to inform policy and practice.

To illustrate the dearth of region-specific research on youth, we conducted a Medline search in January 2013 using the following key words “adolescent*” OR “youth*” with the following limits activated: Humans, Journal Article, English, published in the last 5 years, and Field: Title/Abstract. This search yielded 38415 hits. Adding the medical subject heading (MESH) term “Africa South of the Sahara" reduced the number of hits to 867 (2.3% of the original hits). Although the search is likely biased in terms of focus (medical or public health fields) and the database likely omits a substantial proportion of peer-reviewed journals published by SSA-based institutions, these findings serve as a useful illustration of the limited pool of literature on youth health in SSA.

At country level, nationally-representative data on youth in SSA are primarily gathered through Demographic and Health Surveys (DHSs), which focus on youth and adults aged 15–49 (for women) and 15–54 (for men). This means that early adolescents aged 10–14 are systematically excluded from these surveys [58]. Moreover, DHSs often focus on reproductive and sexual health and are cross-sectional in nature and thus offer limited opportunities for following up youth development in a long-term perspective that could help disentangle causal pathways. Some countries have also implemented the Global School-based Student Health Survey (GSHS) conducted in partnership with the WHO. The GSHS provides useful data on 13–15 year olds but unfortunately is restricted to school-going children in a limited number of countries.

Overall, there are few SSA-based long-term studies on youth development that can clarify linkages between health and the social, political, and economic contexts that define the lives of African youth. However, there are several longitudinal studies in the region that have examined diverse aspects of young people’s health and wellbeing: the Transitions to Adulthood project (TTA), conducted in two informal settlements in Nairobi (Kenya) [37,59,60]; and, in South Africa, the Cape Area Panel Study (CAPS) [61-63] and the Birth-to-Twenty Study (BT20) [64,65], among others. These studies provide a good example of the types of large-scale, longitudinal studies that are needed to better understand the drivers and determinants of young people’s health in SSA. For example, the TTA study made important contributions in areas often neglected in research including youth resilience [66], problem behaviors including substance use [60], and the timing and sequencing of key markers of transitions to adulthood [59]. Likewise, the BT20 study has generated evidence on ethnic differences in bone mass and physical activity; and the impacts of exposure to violence on emotional and social adjustment in children, among others [64].

### Remedying limited research capacity on young people’s health and wellbeing

Developing high-quality research capacity on young people’s health is critical if countries in the region are to develop evidence-based policies and programs to address their needs. However, SSA suffers from limited investments in research and scientific output. Unlike other regions in the world which have several centers of research excellence focusing on young people’s health, the region generally lacks strong research programs that draw together the diverse disciplines needed for a comprehensive understanding of the drivers of young people’s health. Possible ways to address this gap include strengthening inter- and multi-disciplinary research training programs on young people’s health in African universities and the development of south-south and south–north collaborations with institutions having longstanding research experience on young people’s health. While space will not permit us to systematically review current research training opportunities and the lessons they present, Bates and colleagues [67] highlight a variety of approaches to address the shortcomings of research training in African universities, particularly at the doctoral level. These approaches include investments to improve access to electronic resources, improvements in internet connectivity, improvements in the quality of supervision, expansion of the pool of supervisors through collaborations with institutions, and formal skills training on various aspects of research including scientific writing. Several programs to enhance research capacity at African universities in a diverse range of fields [53,68] provide a good framework for addressing the shortcomings of research training.

With regard to the development of research collaborations to build a critical mass of researchers on youth health, the INDEPTH Network serves as an illustration of how like-minded institutions can come together to build a critical mass of researchers able to address youth health. The Network is currently leading discussions on the development of multi-site, multi-country longitudinal research on adolescent transition into adulthood in SSA, with emphasis on young people’s sexual and reproductive health that will be nested on participating health and demographic surveillance sites. This collaborative research undertaking is also expected to provide a platform for researchers to engage with global experts on young people’s health in the region. Some funding organizations are also making important investments in research in adolescent health globally. While few of these research grants are received and led by African researchers based in Africa, they present potential for building local capacity to conduct research on youth health. The creation of a regional association with special focus on young people’s health may be another avenue to bring together researchers, policy makers and program staff working on youth health and development in the region. Creation of such an organization would foster greater attention on young people’s health in the region and provide an opportunity to advocate for services, policies, and research on youth health. One possible approach to developing such an organization would be to initially seek charter membership in organizations such as the International Association for Adolescent Health, or the Society for Adolescent Health and Medicine.

Although a substantial pool of literature on youth health issues published by African scholars may exist in local African and international journals, this literature may be largely unavailable to other researchers, particularly those in Africa. For example, the Journal of Child & Adolescent Mental Health, which is published in association with the South African Association for Child and Adolescent Psychiatry and Allied Professionals, requires a subscription fee. The cost of accessing articles with subscription fees is likely prohibitive for many Africa-based researchers. The creation of an open-access journal focusing on adolescent and youth health in Africa that is supported by an international team of scholars may be one approach to ensure the visibility of this work. Local African journals must also raise their international visibility by ensuring that they are indexed in major databases such as Medline and EBSCO, among others.

## Summary

For the sub-Saharan African region to take advantage of the dividends of a growing youthful population, the region must raise her capacity to generate rigorous scientific evidence to inform policies and programs designed to improve the health and wellbeing of her young people. This means that governments should not only encourage and fund research on young people’s health and development but also actively use evidence generated to inform policies and programs geared towards the youth. Researchers also need to develop innovative ways to reach out to national authorities, policy makers and key stakeholders who will use their evidence in their operations in the continent. Finally, funding agencies and governments should support research on typically under-researched areas of young people’s health in SSA including mental health, injuries, and non-communicable diseases.

## Competing interests

The authors declare that they have no competing interests.

## Authors’ contributions

CWK prepared the journal manuscript. COI and DB reviewed and edited the manuscript. All authors read and approved the final manuscript.

## Pre-publication history

The pre-publication history for this paper can be accessed here:

http://www.biomedcentral.com/1472-698X/13/11/prepub
